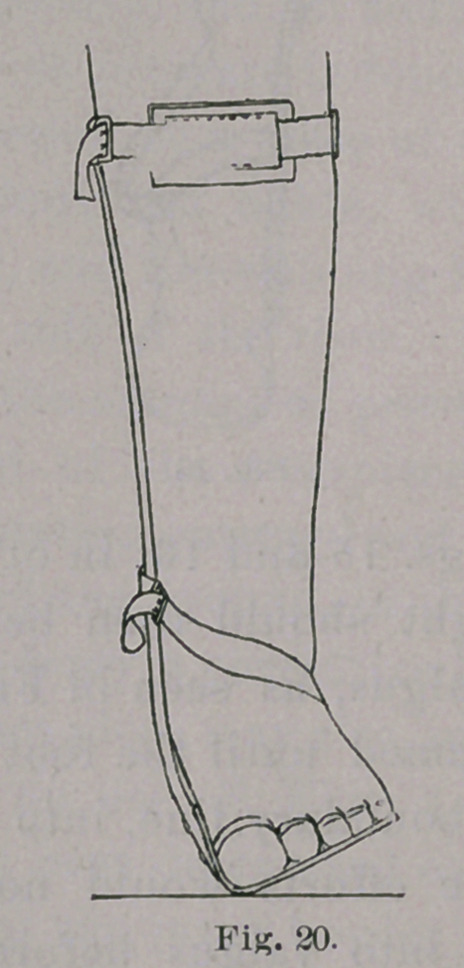# The Weight of the Body in Its Relation to the Pathology and Treatment of Club-Foot1Read before the American Orthopedic Association, New York, September 21, 1892.

**Published:** 1892-10

**Authors:** A. B. Judson

**Affiliations:** Orthopedic Surgeon to the Out-Patient Department of the New York Hospital; 38 East Twenty-fifth Street


### THE WEIGHT OF THE BODY IN ITS RELATION TO THE PATHOLOGY AND TREATMENT OF CLUB-FOOT.[1]

BY A. B. JUDSON, M. D.,

Orthopedic Surgeon to the Out-Patient Department of the New York Hospital.

I DESIRE to present a few thoughts, of an extremely practical kind, relating to the treatment of talipes equino-varus. Beginning with congenital club-foot, it is well to bear in mind that there is a vast difference between a child recumbent and a child walking. While the child is in arms the case is yet free from the complications and difficulties caused by the falling of the weight of the body on the

1. Read before the American Orthopedic Association, New York, September 21, 1892.

deformed foot. These twelve months, more or less, are the most important year in the history of the case, because, in this period, the foot is to be changed so that, when the child begins to walk, the use of a slight walking-brace, exerting only a moderate degree of force, will convert the weight of the body from a deforming to a correcting agent. During these months of recumbency, with the weight of the body out of the way, with all the tissues soft and

Fig. 1.

Fig. 2.

formative, and the foot more than doubling in size with the growth of the child, there is every reason to expect to succeed in what we undertake, provided time enough be given to the case, and faithful attention to the details.

The apparatus which I have conveniently used to effect this reduction, before the child learns to stand, is a simple retentive splint, which acts as a lever, making pressure on the outer side of the foot and ankle, at A, in Figs. 1 to 4, inclusive, and counter-pressure at two points, one on the inner side of the leg, at B, and the other at the inner border of the foot, at C. It is advisable to keep in mind that this simple instrument is a lever, because, if we know that we are using a lever, with its three well-defined points of pressure, we can make the apparatus more efficient than if we view it, in a more general way, as an apparatus for giving a better shape to the foot.

Fig. 3.

Fig. 4.

I use a little brace made of sheet brass, doing the work with a few simple tools. An advantage of doing the work one's self is that there is no room for doubt as to where the blame lies if the apparatus does not work well. Two curved disks, B and C, Figs. 3 and 4, are riveted to a shank, D, and thus is formed that part of the brace which applies the two points of counter-pressure, while, on the other hand, the point of pressure is brought into action by a third disk, or shield, A, which is drawn tightly against the outer side of the foot and ankle, and held in place by a strip of adhesive plaster, E, which includes the limb and the piece which connects the two disks, B and C. The disks are lined with two or three thicknesses of blanket, easily renewed when necessary, with needle and thread. These braces are so cheap and easily knocked together that it is nothing to apply new and larger ones, using heavier material for the shank as the child grows. In general, three sizes will be enough, the shanks being 12 gauge, $\frac{3}{8}$ inch wide, 14 gauge, $\frac{1}{2}$ inch wide, and 16 gauge, $\frac{5}{8}$ inch wide. The disks are conveniently made from 22 gauge, $1\frac{1}{4}$ inches wide. The rivets are copper belt rivets, No. 13. A lip turned on the edges of the disks, with the flat pliers, gives stiffness to the thin brass and protects the skin from the rough edge. If more easily obtained, tin disks, light bars of iron or steel, and ordinary iron rivets, would doubtless answer.

The brace is applied with three strips of adhesive plaster. The upper and lower pieces, F and G, Fig. 4, are simply to keep the apparatus in place, which they do effectively if ordinary gum plaster is used, while, by drawing the middle strip, E, tightly over the shield, and straightening the brace from time to time, the deformity is gradually and gently reduced. At each re-application the brace is made a little straighter than the foot at that stage. This may readily be done by the hands, and then the adhesive strip is to be tightened over the shield, till the shape of the foot agrees with that of the brace. After a few days the brace is to be made still straighter, and again re-applied, and made tight till another point of improvement is gained. The brace is applied very crooked at the beginning of treatment, as in Figs. 3 and 4, and is straightened from time to time, and a longer brace applied as the deformity is reduced and the patient grows. It should be removed every week, or two weeks, and an interval of a few days allowed for freedom from the brace, when the mother is advised to manipulate the foot constantly, using as much force as she will in the direction of symmetry. Manipulating the foot during these intervals is of great importance, as cases have occurred in which varus and equinus have been entirely overcome by the mother's hand alone.

By this simple and prosy treatment, carried out systematically and without haste, or violence, or pain, the foot, unless it is a frightful exception, may, with certainty, be changed from varus to valgus. At the same time, the tendo Achillis is lengthened till the position of the foot is near the norme, or at right angles with the leg, as the result of manipulation and giving brace from time to time a partly antero-posterior action. Figs. 3 and 4 show approximately the shape of the brace at the beginning of treatment ; Figs.

Fig. 5.

Fig. 6.

Fig. 7.

Fig. 8.

5 and 6 when the varus is reduced, and Figs. 7 and 8 when valgus has taken the place of varus. The foot, in this latter stage, may not hold itself valgus when left to itself, but, with almost no force, and with one finger, it may be pushed into valgus, and in this condition it must be when the child begins to walk, and then another stage of treatment begins.

When the patient begins to walk, we have a new difficulty. It is now seen that the weight of the body, falling on the tender and ill-formed foot, will, if not properly directed, defeat all our efforts. Let us, for a moment, consider the mechanical environment of the human foot. In the first place, the corporal weight, which the quadruped distributes among four pedal extremities, fall in man upon two. Again, the small floor area covered by the feet, and their slight structure, seem unequal to the task of supporting the towering frame above them, which in some cases almost resembles a pyramid resting on its apex. And when we observe the effect of active locomotion, we see weight and momentum combine in an apparent effort to crush and destroy. And, furthermore, when extraneous weights are added and the strain is prolonged, as in the case of the burden-bearer among savage tribes, or the infantry soldier on a forced march, the endurance of the foot excites wonder. It is not strange that the feet are subject to ailments, to blisters, bunions, ingrowing nails, hallux valgus, hammer toes, loss of the arch, weak ankles, painful affections of the metatarsus, perforating ulcers, osteitis, and the varieties of talipes. The wonder is that they are not permanently disabled soon after walking is begun, and certainly when the adipose tissue of the body takes on the development which accompanies age and good living. The gourmand, Savarin, said that, among the works of creation, the design of the human foot was a conspicuous failure. Considering the immense weight carried by the foot, it is evident, however, that only the most perfect natural adaptation of mechanics has enabled this insignificant member to perform its superlative functions, and that great caution should attend all procedures having for their object its artificial reconstruction.

It is also sufficiently evident that the correction of club-foot by mechanical means, while the patient continues walking, is a problem beset with difficulty. We have, however, a luminous ray of hope and encouragement in the observation that, in talipes varus, there is an important boundary line between deformity and the norme. If the foot is held in some way, now to be considered, on the right side of this boundary line, each step forces it in the direction of valgus, and the increasing weight of the child is a powerful force acting in the right direction, or away from varus, so long as the foot is held, though never so little, looking toward symmetry. It may be said that the child stamps his foot straight. If, on the other hand, the foot is held, or allowed to fall, on the wrong side of this line, though never so little, each footstep is a blow, driving the foot more and more into the varus position.

This point may be illustrated by the hand placed with its ulner border on the table. If considerable pressure be made on the table by the hand so placed, it becomes evident that there is a boundary line between pronation and supination. If the hand is pronated, never so little, additional pressure will force the palm nto pronation, which represents valgus in the foot, and if the hand be supinated in the slightest degree, additional pressure will force the palm into complete supination, which represents the varus in the foot.

By the application of this idea, the weight of the body may be made a beneficent instead of a harmful factor in the progress of a case of talipes varus, and the walking brace should be constructed with this in view. It should be made of steel, and by an instrument maker. One of its functions is to act as a lever, but the leverage is applied not chiefly to overcome the deformity by direct force, as in the retentive brace above described, but to hold the foot on the right side of the boundary line above mentioned, so that the weight of the body may straighten the foot, or overcome the varus in a direct and forcible manner, without general or local inconvenience.

The walking-brace consists, as usual, of leg-band, H, Figs. 9 and 10, foot-piece, I, and upright, J, riveted firmly together. A movable joint at the ankle should be discarded, as it undermines

Fig. 9.

Fig. 10.

the lever by introducing an element of instability and, in this brace, serves no good purpose. Mild steel alone should be used, to facilitate alterations in shape, as point after point of improvement is gained, and to make easy the shifting of buckles and straps, as may be required, all of which may be done by the use of a few simple tools. The upright is to be on the inner side of the leg, as in Fig. 14. The upper part of the brace makes counter-pressure on the inner side of the leg, but it has another important function, in previously neglected cases, which is secured by the steel band passing across the back of the leg, to which are fastened two buckles for the attachment of a piece of webbing, K, in Fig. 9, which passes across the front of the leg. The steel band should make no pressure on the limb as its use is simply to furnish attachment to the buckles. A piece of webbing spanning the front of the leg in this manner, and carrying a pad, performs an important service in cases like the one shown in Fig. 12, in which, from previous neglect, the varus has not been reduced before walking begins. It transfers a part of the weight of the body from the anterior part of the sole of the foot, where it interferes with the correction of the varus, to the upper part of the anterior surface of the leg, where it is powerless to interfere with the treatment. That the weight-pressure thus transferred is considerable, is shown by the callus and bursa which appear where the padded strap crosses the leg near the tubercle of the tibia. This mechanical effect is similar to that of the brace shown in Fig. 11, used in the treatment of paralysis of the muscles of the calf, resulting in talipes calcaneus.

The upper part of the brace is also to be considered in another light, as follows: In previously neglected cases it is well to incline

Fig 11

Fig. 12.

the upright 15° or 20°, or more, backward from the vertical of the foot-piece, as is shown in Fig. 9. Although correction of the equinus is postponed by this inclination of the upright, we are thus enabled to apply a better leverage against the varus, and when the varus is reduced, and the time arrives when the equinus is to be corrected, this backward inclination of the upright is to be lessened from time to time, till the vertical is reached, as in Fig. 10, or till the upright has an inclination forward, allowing the corporal weight to fall more and more on the anterior part of the sole of the foot, and gradually lengthen the tendo Achillis. The vertical upright, Fig. 10, is to be applied at once to patients in whom the deformity has been corrected before walking begins.

We will now pass to a consideration of the other end of the brace, the foot-piece, which is to be made of sheet steel ranging from 18 gauge, for a child learning to walk, to 13 gauge for an adult. It has the usual tread, L, Fig. 13, and riser, M, Fig. 10. The heal-cup is formed by a piece of webbing, N, Fig. 13, passing behind one heel, from the lower part of the upright to a spur, O, Fig. 13, which projects upward from the back part of the outer border of the tread. Viewing the apparatus again as a lever, for the forcible reduction of varus, in a previously neglected case, counter-pressure is made along the inner border of the foot, and on the upper part of the inner side of the leg, while pressure is made by one strap, or more than one, riveted and buckled to the foot-piece and the upright. But one strap is shown, P, in Figs. 13 and 14.

Fig. 13.

Fig. 14.

This will be sufficient in the case of a child whose varus has been corrected before walking begins, but in a previously neglected patient, in whom the varus has yet to be reduced while the child is active on his feet, two, three, or more straps may be added, as shown in Fig. 9, partly encircling the foot, ankle, and leg, the positions of the buckles and the straps being where they will assist most efficiently in opposing the varus and holding the foot in the best position to receive the weight of the body. These parts of the apparatus may beshifted many times with advantage in the treatment of a given case of unusual difficulty, and, in addition, a most efficient agent for applying continuous pressure is found in a strip of adhesive plaster, Q, Fig. 14, sewed to a piece of webbing, R, the plaster partly encircling the foot and ankle, with a single tail or two tails, as may be required, and the webbing being drawn tightly and buckled to the inner side of the riser. This device does more than simply to increase the amount of pressure ; it also keeps the heel down on the tread of the foot-piece and, more important still, it gives the foot a rotation outward and thus directs the sole of the foot forcibly toward the ground, in the best position for making the weight of the body a corrective instead of a deforming force. The riser of the foot-piece may also, in previously neglected and difficult cases, carry an ear, S, Figs. 9, 13 and 14, made of sheet brass, which is to be bent downward over the first metatarso-phalangeal joint, to prevent the inner border of the foot from over-riding the edge of the riser. The foot-piece is to be lined with adhesive plaster, in several thicknesses, if necessary, to prevent rust, and with a piece of leather fastened to the tread and spur with copper rivets, as shown in Fig. 10. In practice, the details demand as much attention as the principles of treatment. The brace is to be applied over the stocking, the strap, R, passing through a hole cut in the stocking, and is hidden by the patient's trousers and shoe.

We will now consider the upright of the brace. It is a flat, tapering bar of mild steel, and, when first applied to a previously neglected case, such as is shown in Fig. 12, should have a curve resembling that of the varus foot. The bar, though sharply curved, as in Fig. 13, should, however, be somewhat straighter than the foot; then the latter is forced manually into its best position. The multiple straps, shown in Fig. 9, should then be buckled and tightened daily till the continuous leverage has partly reduced the varus. The upright bar should then be somewhat straightened, and another point of improvement be gained, the patient in the meantime following his ordinary pursuits without interruption. In due time the upright bar, and the foot itself, will both be straight, as

Fig. 15.

Fig. 16.

seen in Figs. 15 and 16; in other words, the varus will be reduced. The upright should then be bent, from time to time, in the direction of valgus, as seen in Fig. 17, and the persistent and gradual effort resumed until the foot has been pushed, or pulled, or pried, over the boundary line, into the domain of valgus, as seen in Fig. 18. These efforts would not be necessary if the varus had been converted into valgus before the child had learned to stand. In very badly neglected cases this interference of the weight of the body with the treatment may be prevented by the recumbent position, or the use of a high sole on the well foot and the ischiatic or axillary crutch, until the varus has been materially reduced. In all cases, when the child is old enough to be docile, domestic instruction and drill in eversion of the foot, and in the proper

Fig. 17.

Fig. 18.

management of the foot in locomotion, should be a part of the education.

As soon as the foot has reached the valgus shape, whether it be at the moment of learning to walk, or only after prolonged effort in a neglected case, a curious effort will be observed. It will be seen

Fig. 19.

Fig. 20.

that the outer border of the tread of the foot-piece is raised from the ground, as seen in Figs. 19 and 20, and that we have secured, in a convenient manner, the effect which is sometimes sought by building up the outer border of the sole of the patient's shoe. This is a welcome and powerful ally in our attempts to hold the foot in a favorable relation with the weight of the body and the ground.

The walking-brace has been above described as though its chief use were to reduce varus which has become more or less confirmed by the habit of walking on the outer border of the foot. Strictly speaking, such cases should never occur. They are, however, too common and always indicate that the child has been neglected from the period of recumbent infancy, when deformity of this kind is the most easily overcome. If the varus were always corrected before the child learns to stand, then the only use of the walking-brace would be, as shown in Figs. 19 and 20, to gently hold the foot in valgus, so that the weight of the body shall be sufficient to lead the child to grow up with a foot practically normal. As such a child outgrows the brace, a larger one is to be made, and, when three or four years old, the foot will, without the help of the brace, strike the ground so fairly that, for two or three years, all treatment may be suspended. The patient is to be observed from time to time, however, and, as the foot grows in its original inclination to varus, it will, after the lapse of two or three more years, have to be kept in proper position, under the rapidly increasing weight of the body, by a walking-brace adapted to its needs, for another period of two or three years. When the foot is full-grown, it will be shapely in appearance and practically perfect in its ability to perform all the duty of a foot congenitally normal.

Although congenital club-foot has been chiefly kept in mind in the foregoing pages, the views expressed in regard to the influence of the weight of the body are applicable also to talipes varus of paralytic origin. In this affection, at an early stage, and before the foot has lost its flexibility, a simple walking-brace is needed, as in Figs. 19 and 20, to properly direct the action of the weight of the body on the paralyzed foot. At a later period, if this measure has been neglected, and the foot has been allowed to become varus and more or less inflexible, the case will require more attention and probably prolonged effort, with multiple straps and adhesive plaster, to carry the foot across the line between deformity and the norme, to the position in which the weight of the body shall be a correcting and not a deforming force.

38 East Twenty-fifth Street.

## Figures and Tables

**Fig. 1. f1:**
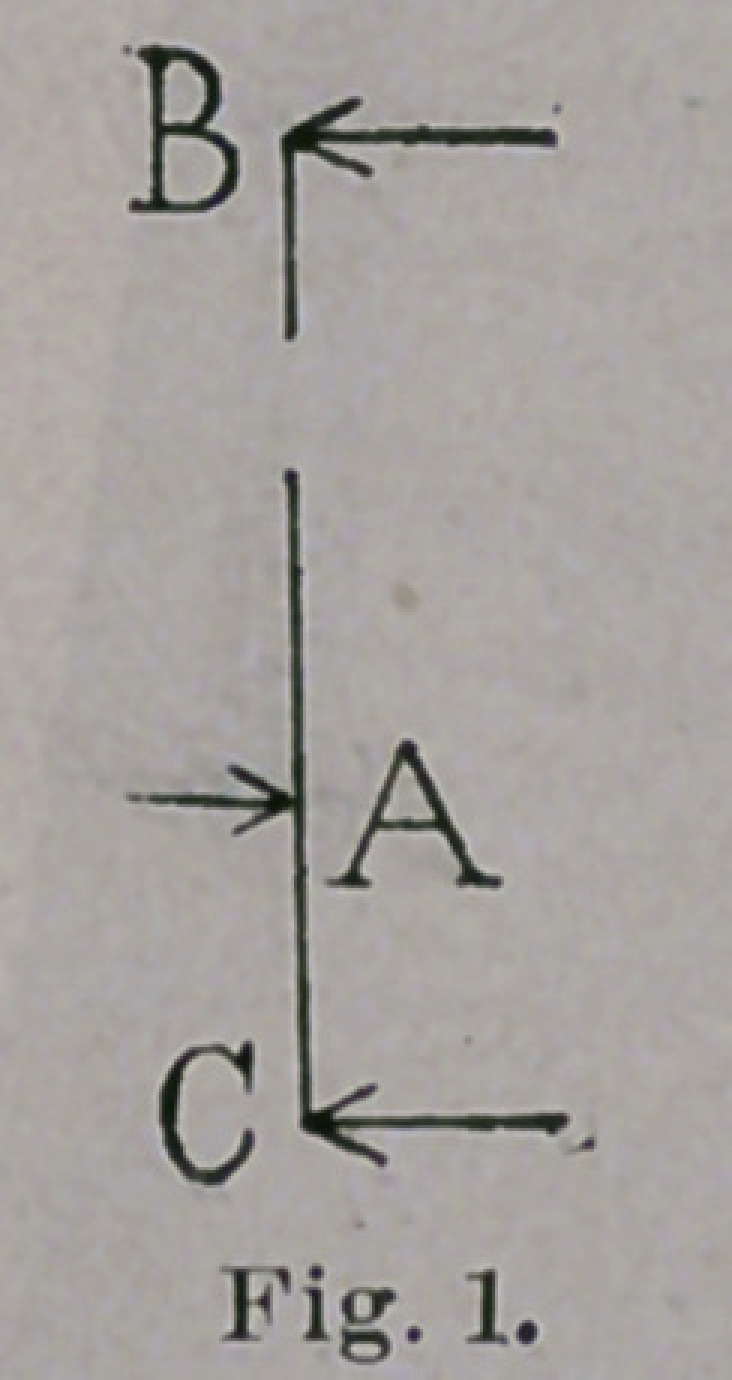


**Fig. 2. f2:**
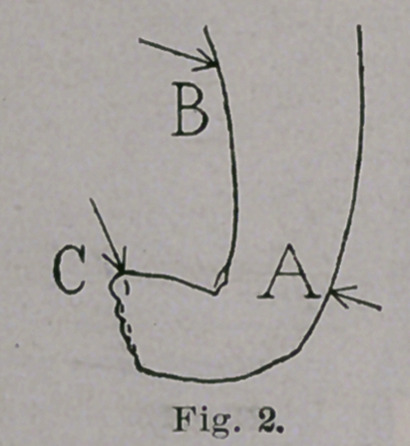


**Fig. 3. f3:**
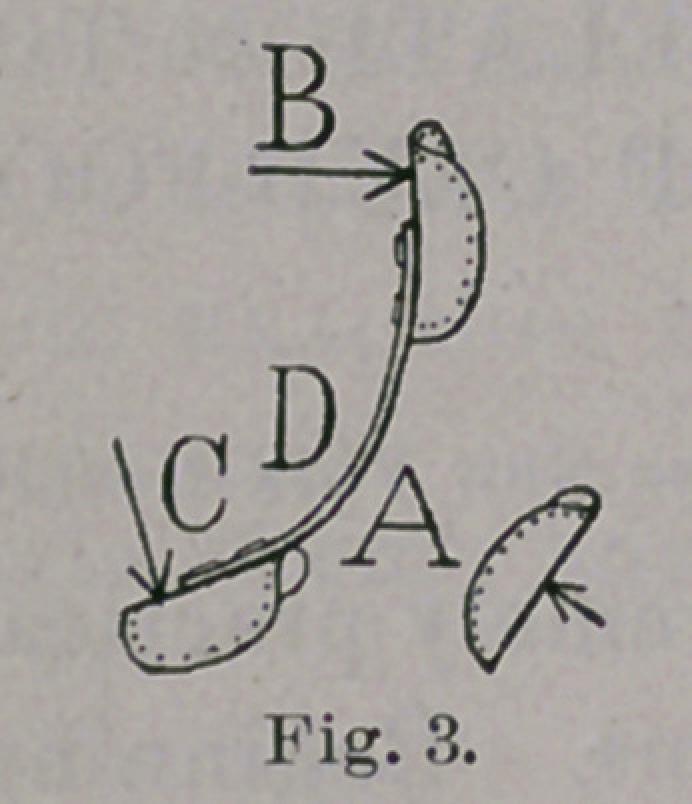


**Fig. 4. f4:**
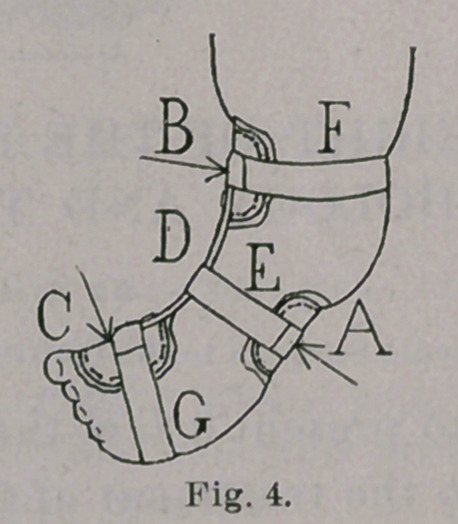


**Fig. 5. f5:**
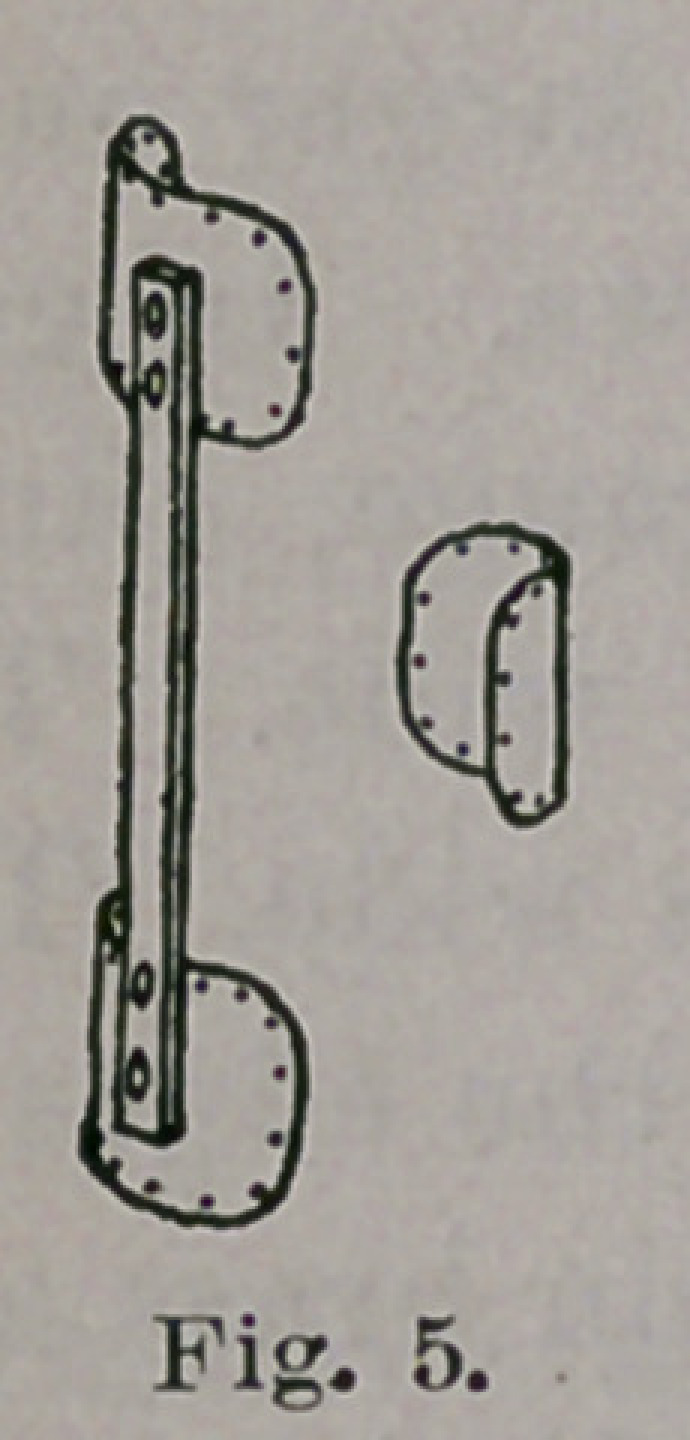


**Fig. 6. f6:**
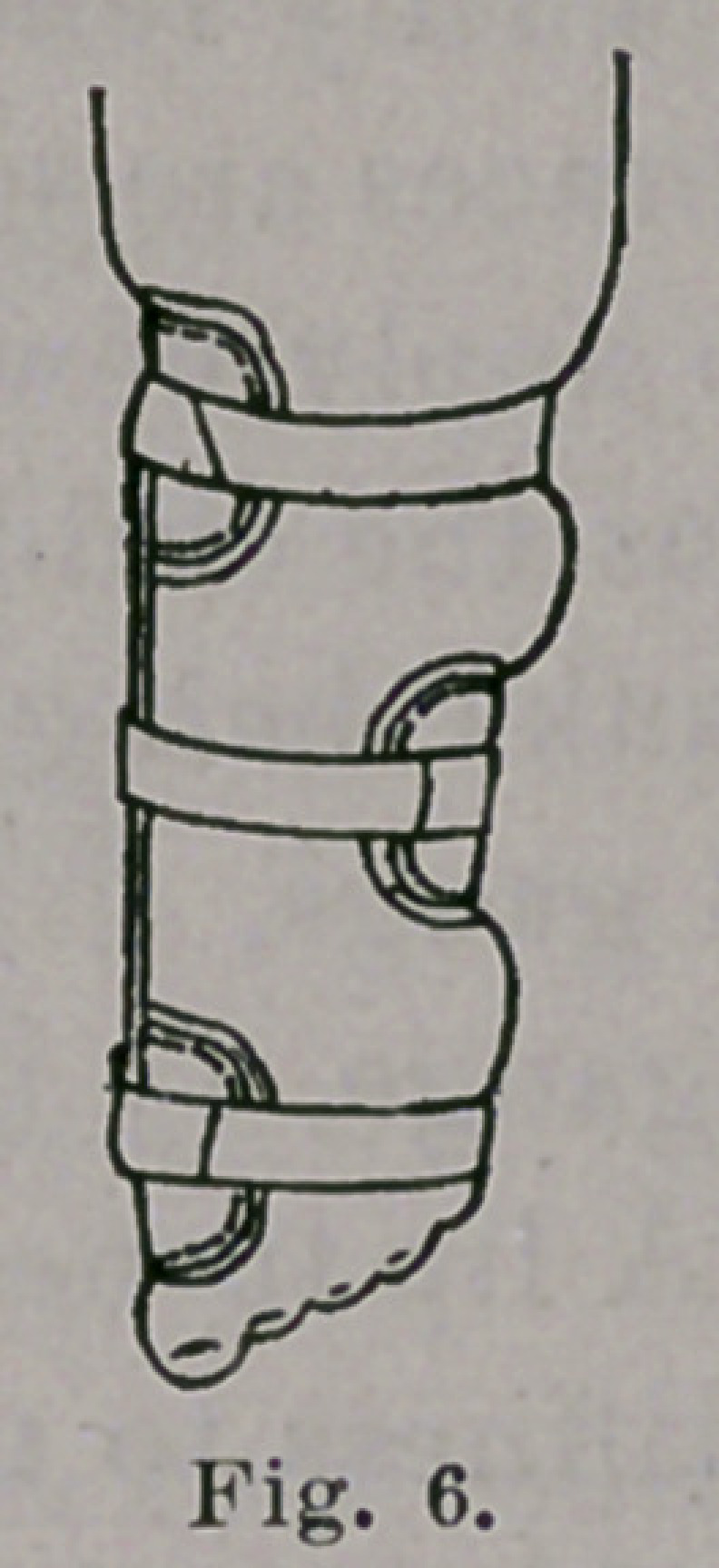


**Fig. 7. f7:**
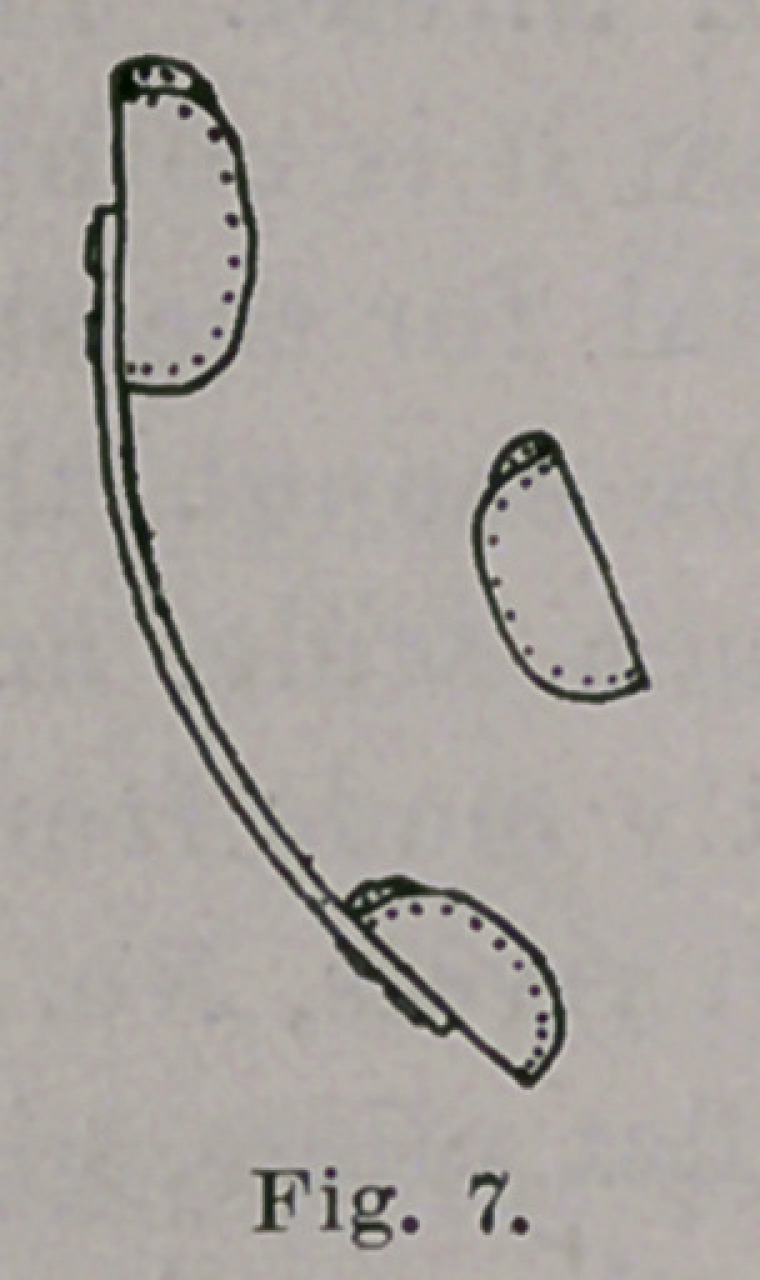


**Fig. 8. f8:**
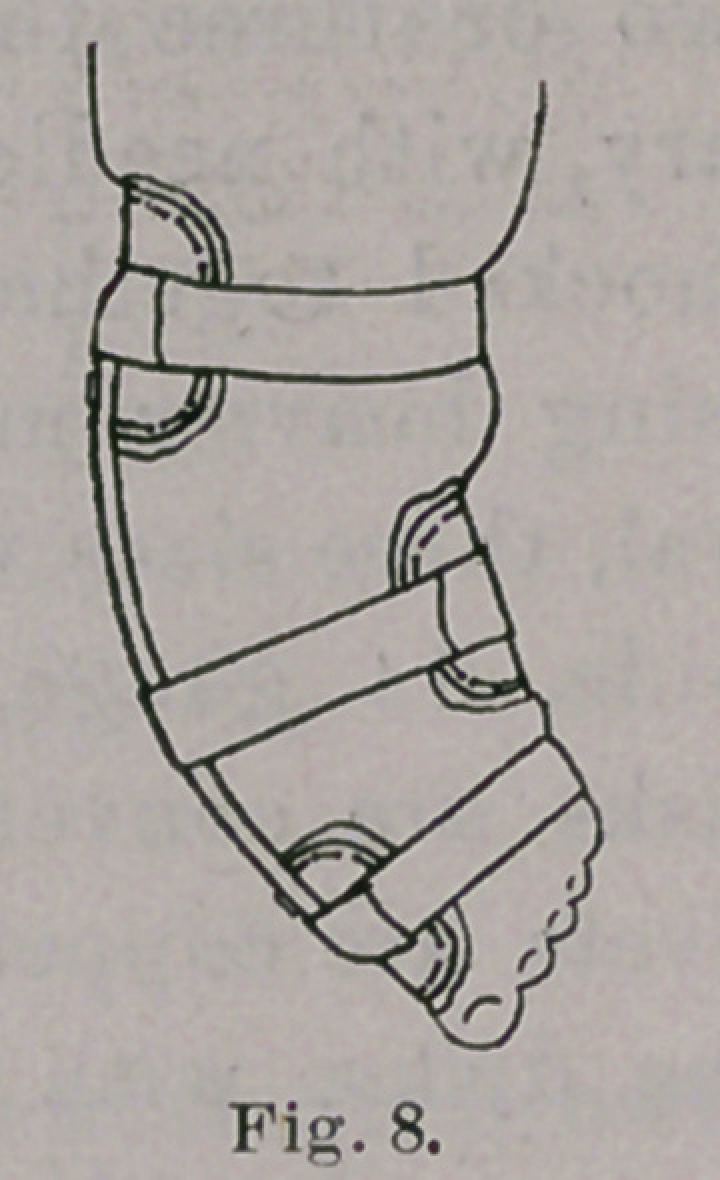


**Fig. 9. f9:**
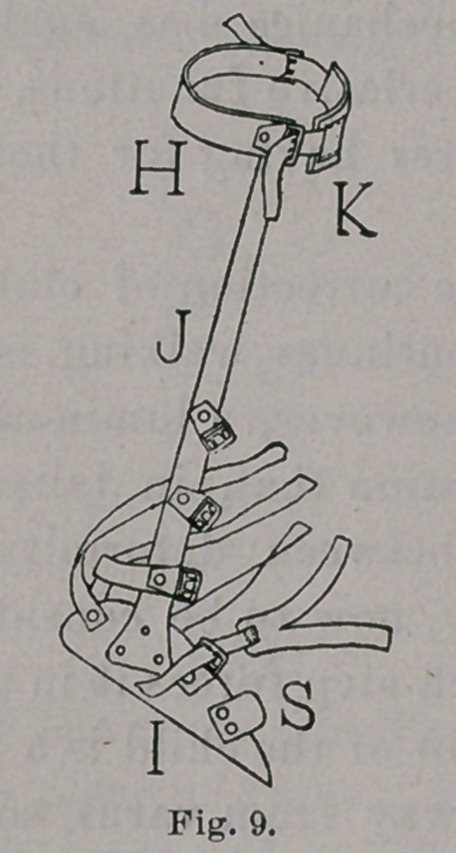


**Fig. 10. f10:**
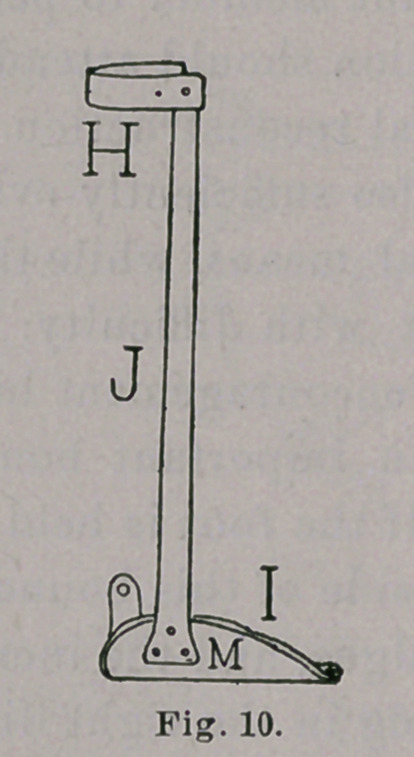


**Fig 11 f11:**
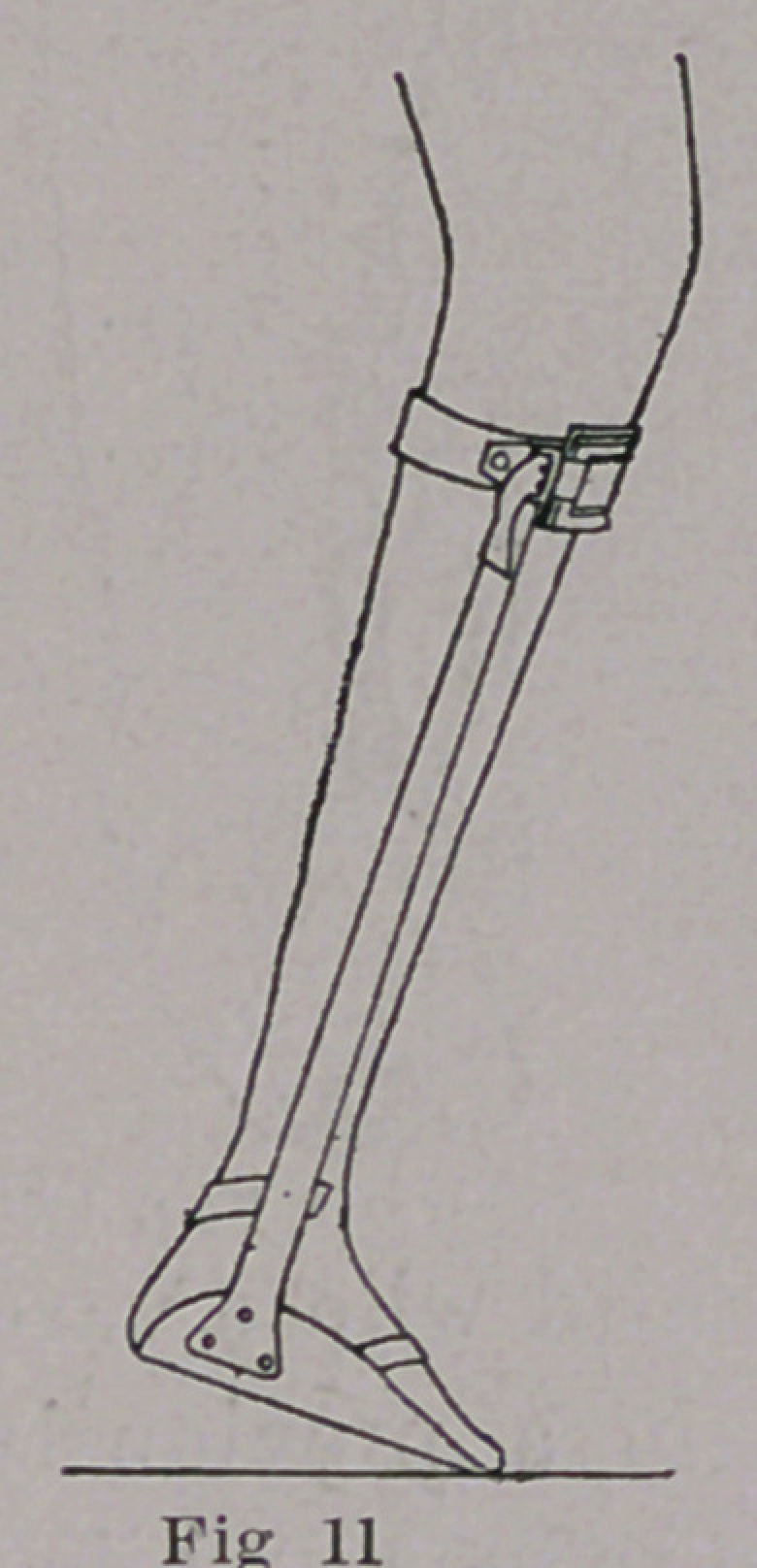


**Fig. 12. f12:**
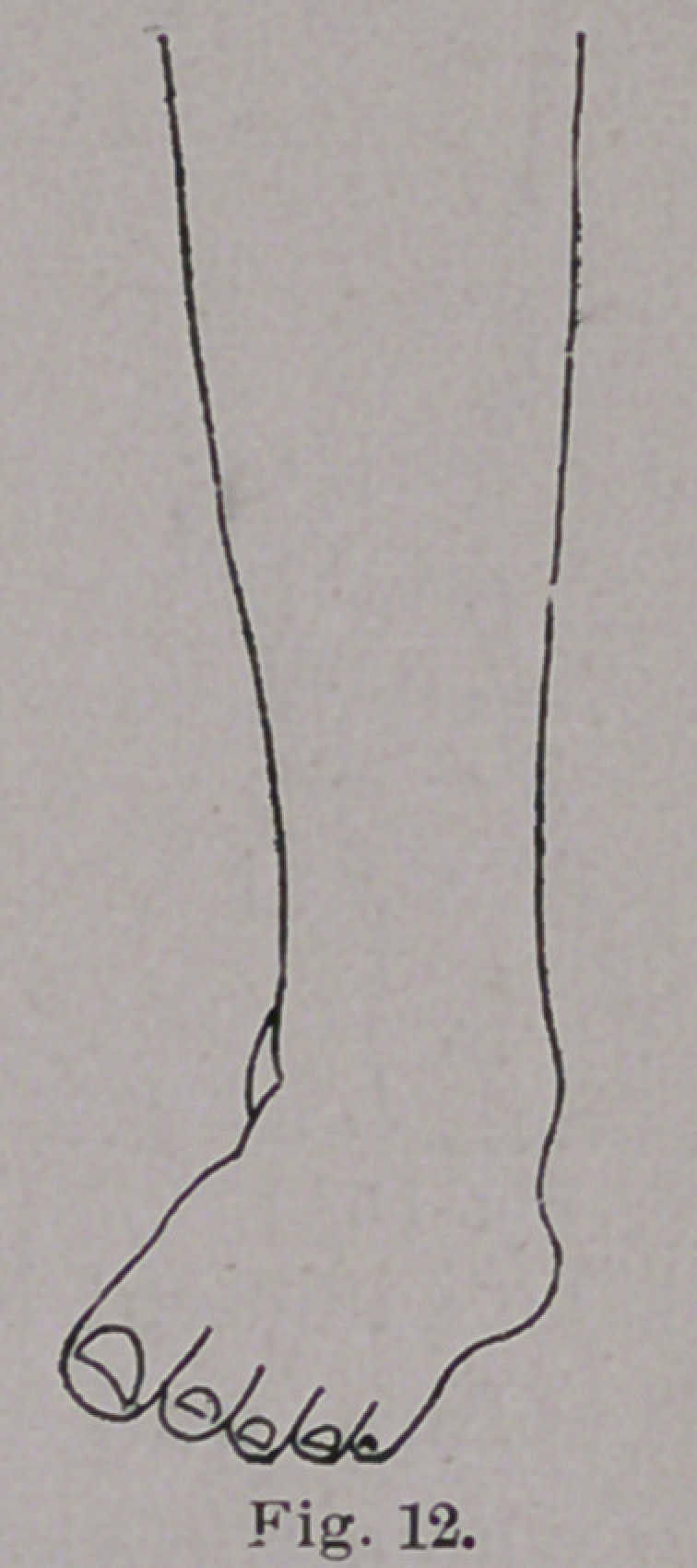


**Fig. 13. f13:**
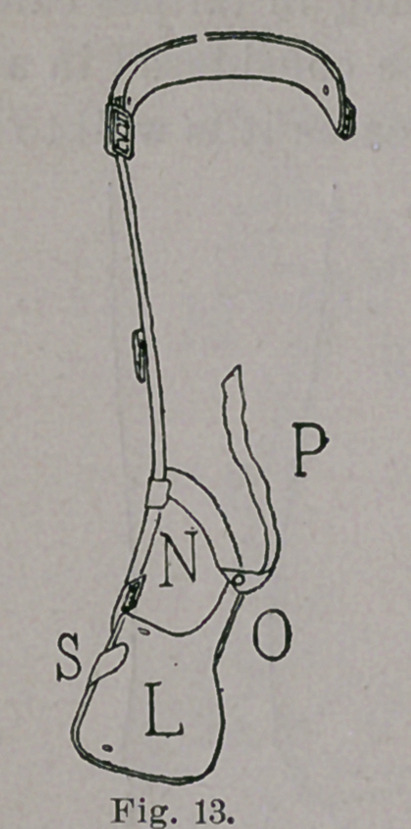


**Fig. 14. f14:**
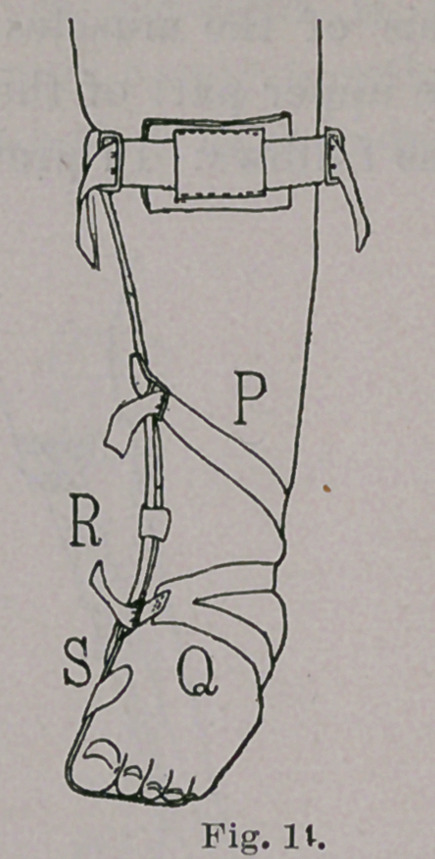


**Fig. 15. f15:**
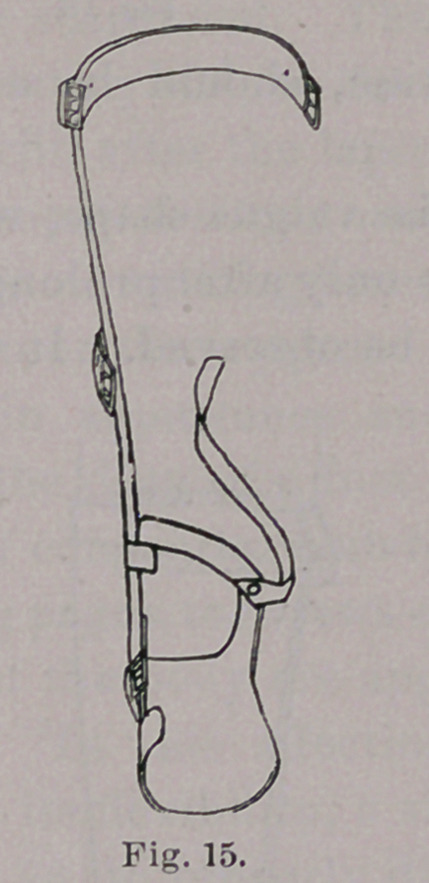


**Fig. 16. f16:**
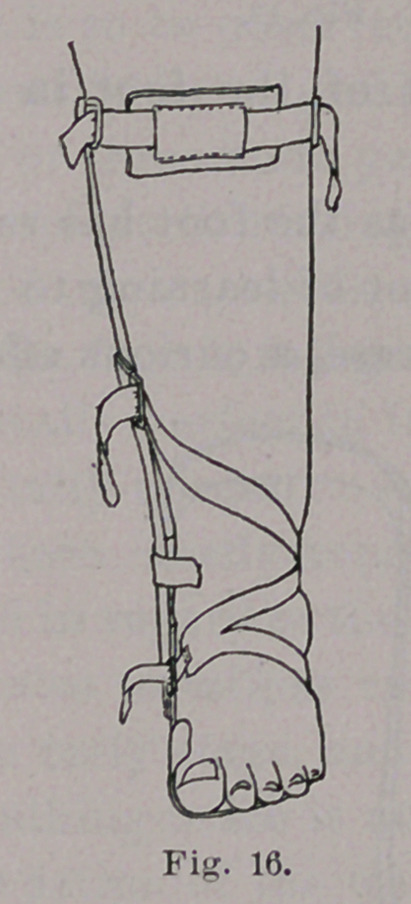


**Fig. 17. f17:**
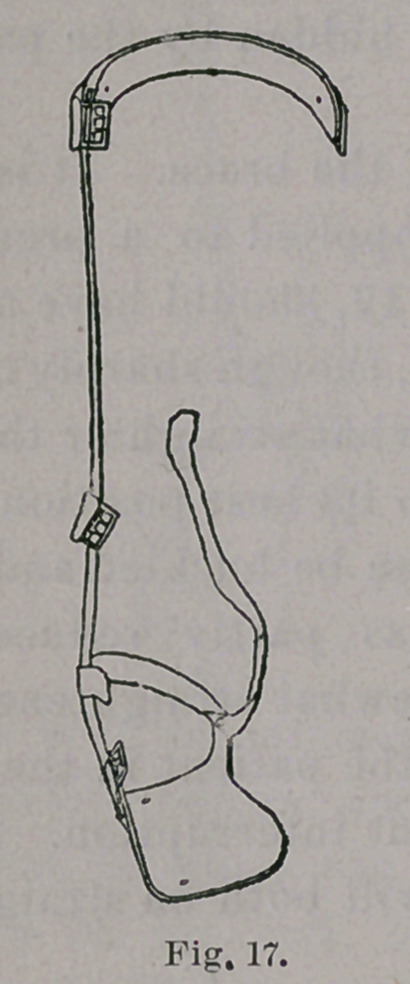


**Fig. 18. f18:**
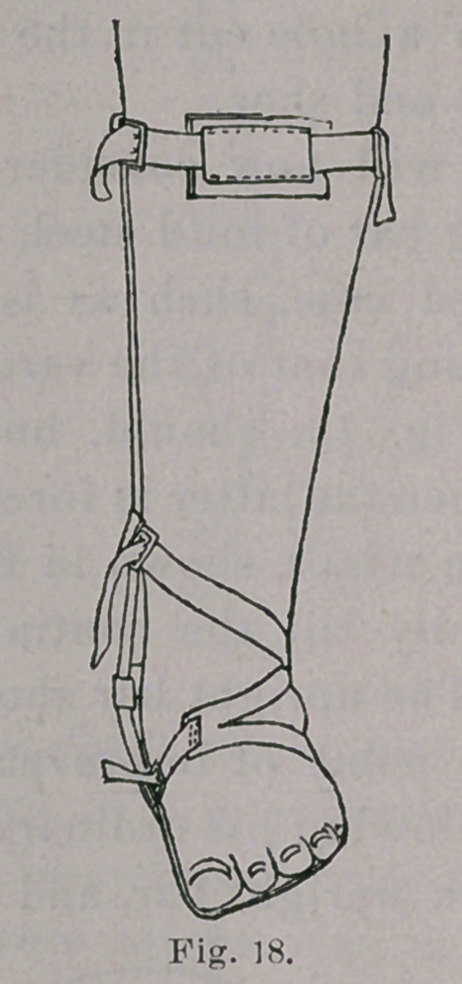


**Fig. 19. f19:**
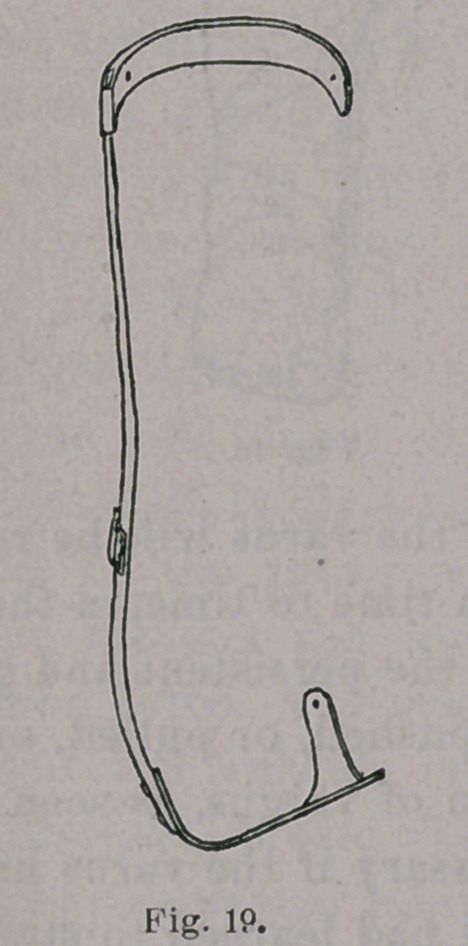


**Fig. 20. f20:**